# Willingness to receive lung cancer screening when engaged in community-based locations

**DOI:** 10.1016/j.xjon.2026.101942

**Published:** 2026-06-25

**Authors:** Raphaela Varella, Maya Fajardo, James Moss, Maguel Ross, Cameron Craig, Ivan Kyagaba, Kyra Goeller, Luv Thakkar, Alqasim Elnaggar, Alejandro Damian, Olivia Aspiras, Todd Lucas, Ikenna Okereke

**Affiliations:** a Wayne State University School of Medicine, Detroit, Mich; b Charles Stewart Mott Department of Public Health, Michigan State University, Flint, Mich; c Department of Surgery, Henry Ford Health, Detroit, Mich

**Keywords:** lung cancer, community engagement, lung cancer screening, attitudes, disparities

## Abstract

**Objective:**

Lung cancer is the leading cause of cancer deaths. Low-dose computed tomography (LDCT) screening enables early detection and reduces lung cancer mortality. Yet, less than 10% of eligible individuals participate in LDCT. This study leveraged community connections to measure willingness to undergo lung cancer screening among a diverse population.

**Methods:**

Black (n = 437) and White (n = 122) Americans were recruited from community-based locations (eg, barbershops, salons, libraries) in a large city. Participants completed a survey measuring willingness to undergo lung cancer screening if recommended (1 = not at all likely, 10 = very likely). They also answered questions about individual-, provider-, and structural-level screening barriers: medical mistrust (trust in health care system to deliver accurate screening results; 1 = very little trust, 10 = significant level of trust), perceived provider recommendations (how well primary care doctors promote screening in their community; 1 = very poorly, 10 = extremely), and financial accessibility (likelihood of insurance coverage of screening; 1 = very unlikely, 10 = very likely).

**Results:**

Mean age was 38.59 years; 53.6% were female. Screening willingness did not differ by race (Black American: 7.27 ± 3.04 vs White American: 7.21 ± 2.86). Black Americans perceived greater insurance coverage for screening (6.97 ± 3.01 vs 6.43 ± 2.84; *P* = .07) and lower trust in screening accuracy (6.73 ± 2.56 vs 7.20 ± 2.19; *P* = .045) than White Americans. No racial differences were found for provider recommendations (Black American: 4.95 ± 2.83 vs White American: 5.08 ± 2.26). Screening willingness was positively associated with financial accessibility (*P* < .01), trust (*P* < .01) and provider recommendations (*P* < .01).

**Conclusions:**

Screening willingness is shaped by financial accessibility, provider recommendations, and trust among both Black and White Americans. Mistrust emerged as a prominent barrier for Black Americans, whereas concerns about insurance coverage were a barrier among White Americans. Efforts to increase screening uptake should use a multilevel approach, with attention to needs across racial groups. The successful recruitment of participants from community-based locations indicates that residents are receptive to information about lung cancer screening outside clinical settings, highlighting opportunities to expand screening education and efforts to these locations to reach diverse populations.

**Figure:**
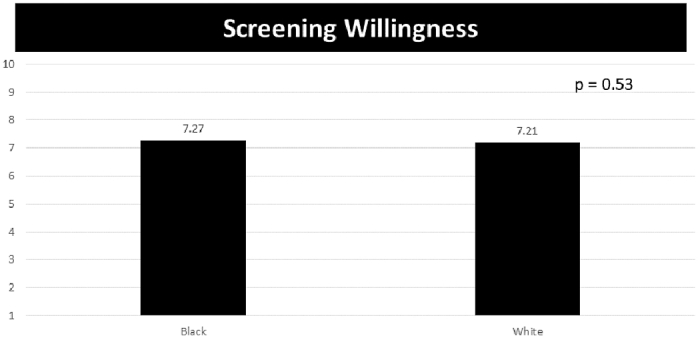
Screening willingness in survey participants.

**Central Message**

Willingness to undergo lung cancer screening is shaped by financial accessibility, provider recommendations, and trust. Race does not appear to be associated with willingness to receive screening.

**Perspective**

Lung cancer is the leading cause of cancer deaths in the United States in both men and women. Less than 10% of eligible individuals undergo lung cancer screenings. Survey participants demonstrated a willingness to undergo screening but perceived significant barriers to care. There was no racial difference in willingness to be screened.

Lung cancer is the leading cause of cancer-related deaths in the United States.^1^ Recommendations have been made to increase rates of lung cancer screening in recent years.^2^^,^^3^ The use of low-dose computed tomography (LDCT) screening for lung cancer in specific cohorts has been compelling. For high-risk patients, the lung cancer–specific mortality rate for those who underwent LDCT was significantly lower than those that did not get any type of screening.^4^^,^^5^ Despite this evidence, efforts have not been successful in establishing widespread uptake in screening rates among eligible patients.^6^^,^^7^ Currently, the rate of screening among individuals who meet the guidelines is less than 20%.

The current gaps to obtaining lung cancer screening are in specific steps in the overall process involved in receiving the scan. Inaccurate or nonspecific smoking history can affect participation, because individuals may underestimate their exposure or misunderstand the threshold.^8^^,^^9^ Second, providers may not counsel eligible patients about lung cancer screening for multiple reasons.^10^^,^^11^ In addition, the currently required shared decision-making visit may seem perfunctory to patients and dissuade them to engage further in screening.^12^ Finally, there is likely a smoking-related stigma that dampens overall interest in lung cancer screening.^13^^,^^14^

There is a significant racial disparity in the rate of lung cancer screening, as Black Americans are less likely to undergo lung cancer screening compared with White Americans.^15^ Multiple factors are likely associated with this disparity, including various social determinants of health. These determinants are numerous and include considerations such as financial insecurity, transportation difficulties, and lack of medical insurance.^16^, ^17^, ^18^ These factors can increase the obstacles faced by Black Americans and subsequently decrease the likelihood of undergoing lung cancer screening.

Another major reason that Black Americans are less likely to be screened is related to the uneasiness and lack of trust with the health care system. Injustices such as the Tuskegee Syphilis Study^19^ and structural discrimination related to maternal health^20^ have created a sense of mistrust and discomfort for many Black Americans regarding medical institutions. To counteract this distress and anxiety, community engagement by medical institutions has been associated with increased satisfaction and willingness by individuals to participate in measures such as vaccination.^21^

Previous studies that have investigated willingness to undergo lung cancer screening have not focused on the role of community engagement and the context in which individuals are approached. Although many studies have focused on a cohort of screening-eligible individuals, there has been growing interest in the perceptions of pre-eligible individuals. Previous literature has raised the idea that supporting intention formation among pre-eligible people may strengthen future screening uptake and improve rates of screening.^22^ Our goal was to investigate the effect of engagement of individuals in community-based locations outside the hospital and to measure racial disparities when approached in these community-based areas.

## Methods

### Survey

**Figure 1:**
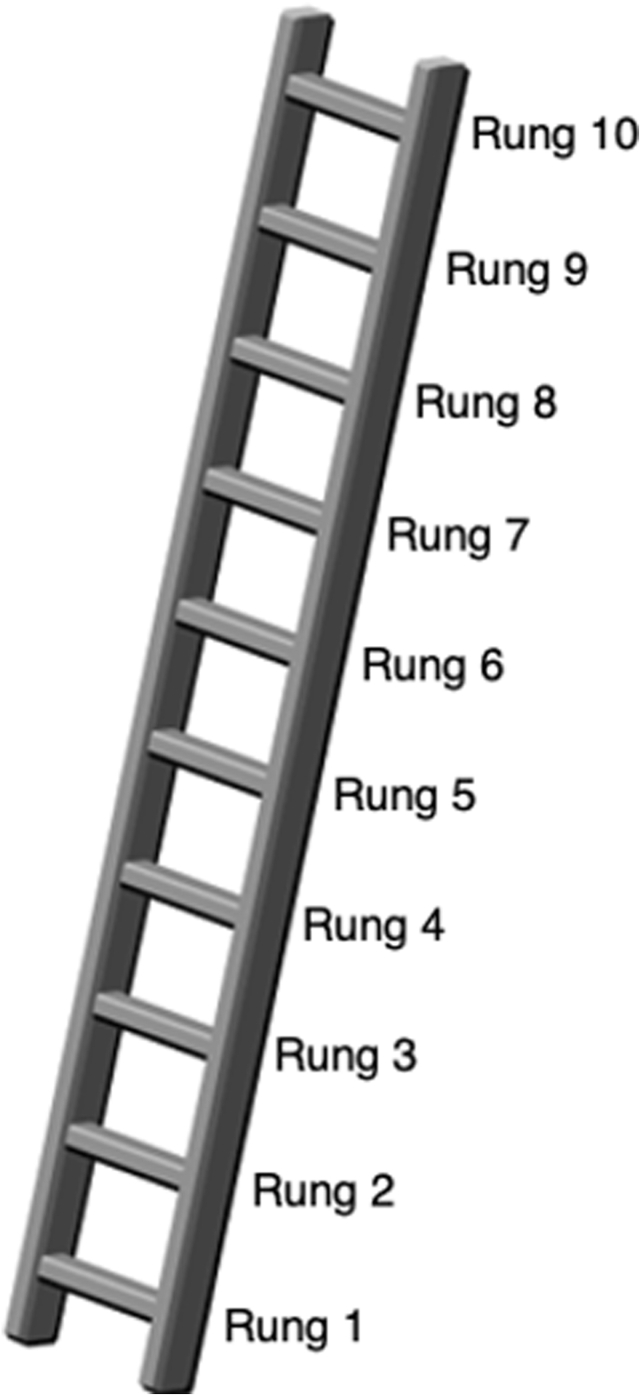
Social ladder.

Institutional review board approval was obtained before we conducted the study (institutional review board no. 17851, January 7, 2025). The survey is presented in Online Data Supplement. Participant sociodemographic information was obtained, including age, sex, race and perceived social standing. Perceived social standing was measured using the MacArthur Scale of Subjective Social Status,^23^ displayed as an image of a ladder with 10 rungs (Figure 1). Each rung measured the perceived social standing, with the bottom rung representing the lowest social standing and the top rung representing the highest social standing of the respondent.

Of present interest, the survey included a measure of willingness to undergo lung cancer screening if recommended (*How likely are you to pursue lung cancer screening if recommended?* 1 = not at all likely, 10 = very likely), as well as items measuring various individual-, provider-, and structural-level screening barriers. Participants answered questions about their perceived comfort in recommendations from their individual provider (*How well do you believe that primary care doctors promote lung cancer screenings among patients in your community?* 1 = very poorly, 10 = extremely well), structural-level screening barriers and mistrust of screening (*What level of trust do you hold in the health care system to return accurate lung cancer screening results?* 1 = very little trust, 10 = significant level of trust), and financial accessibility (*How likely do you think that your medical insurance would cover lung cancer screening if recommended?* 1 = very unlikely, 10 = very likely).

### Survey Distribution

Black and White Americans were recruited from community-based locations in a large metropolitan city. The city currently has a population that is 73% Black American. Potential participants were approached by research assistants, who provided a brief verbal description of the study as well as some general information about lung cancer. Adults aged 18 and older were eligible to participate.

### Procedure

Consent was obtained verbally and as the initial step of the online consent, including consent for publication. After providing consent, participants completed a survey administered electronically on an iPad. Before completing the survey, participants were given brief education on lung cancer and lung cancer screening. Specifically, participants were told of (1) lung cancer etiology, (2) major risk factors for lung cancer development, (3) current guidelines for lung cancer screening, (4) the process of lung cancer screening including obtaining LDCT, and (5) the literature showing a reduction in mortality for eligible adults who underwent lung cancer screening.

To address potential language and digital literacy barriers, research assistants were available to provide support as needed. Assistance for participants included reading survey questions aloud, clarifying question wording when requested and entering responses on behalf of the participant at the community-based location. Upon completion of the survey, participants provided their email to receive a $5 electronic gift card as compensation for their time.

### Community Locations

Community locations were selected to engage diverse populations and reach individuals who may have limited access to health care settings. Owners and managers of the chosen establishments or events were then contacted via phone or email. Permission was obtained for the study team to recruit for the survey at each location. Once approval for recruitment was obtained, members of the study team coordinated with the locations about days and times for survey recruitment. Survey administration was carried out in teams of approximately 2 to 4 researchers at each community location, depending on the size of the location. Team members brought multiple iPads to the location for greater simultaneous completion to allow for easier flow at high-traffic events and large gatherings. Recruitment occurred during typical daily operations at locations as well as during city-specific local events where significant attendance was expected. Specific events included community-based cultural events. The research team also hosted surveying events at local Detroit churches upon the conclusion of Sunday morning religious services.

### Statistical Analysis

Racial differences in individual-, provider-, and structural-level screening barriers and in screening willingness were analyzed using independent samples *t* tests. Bivariate correlations were conducted to assess the association between each barrier and screening willingness.

## Results

### Community Locations

In all, 41 locations across Metro Detroit were visited at least once for survey recruitment. These locations included community-based cultural events, popular outdoor spaces, faith-based centers, local businesses, and academic institutions (Table 1).

**Table 1:** Community locations

Gymnasiums
Athletic centers
Barber shops
Hair salons
Churches
Libraries
Pride festival
College campus
Wellness outreach fairs
Farmers markets
Restaurants
Belle Isle park
Eastern Market
Wedding reception
Detroit Riverwalk
Vibes with the Tribes Native American Festival
Hart Plaza
Baby shower

### Participants

A total of 559 participants, including 437 Black and 122 White Americans, were surveyed. Mean age was 38.59 ± 16.70 years. Female patients comprised 53.6% of the total.

### Social Ladder

**Figure 2:**
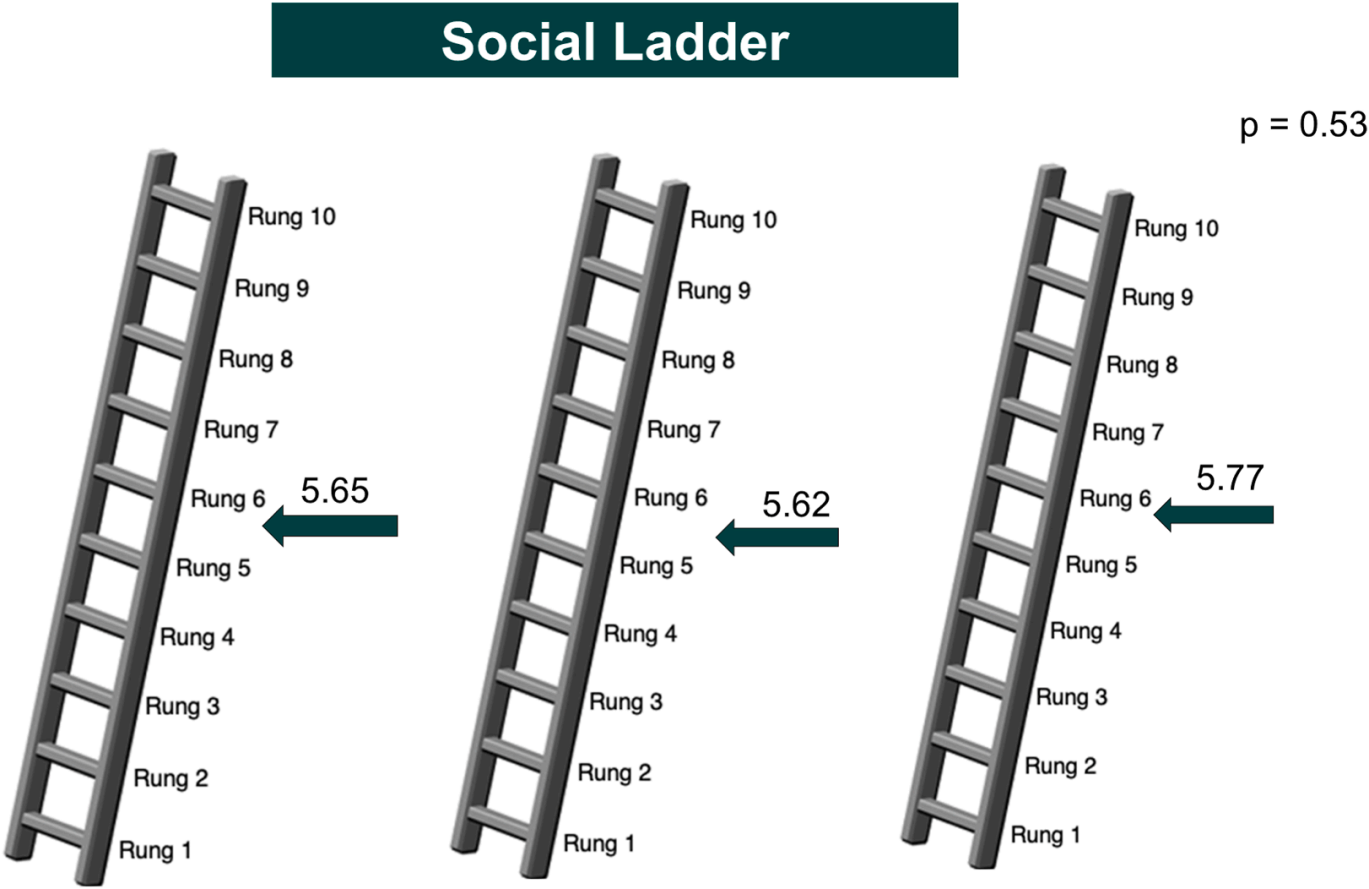
Comparison of social standing between Black and White participants.

Across the entire cohort, survey participants reported a moderate perceived social status (5.65 ± 2.37). When comparing Black Americans with White Americans, we found there was no significant difference in perceived social standing (Figure 2; Black American: 5.62 ± 2.46 vs White American: 5.77 ± 2.03, *P* = .53).

### Screening Willingness

Willingness to be screened was generally high in the entire cohort. Results of the independent samples *t* test showed that screening willingness did not significantly differ by race (*t*[557] = .19, *P* = .853; Black American: 7.27 ± 3.04 vs White American: 7.21 ± 2.86). When we examined the entire cohort, screening willingness was positively associated with financial accessibility (*r* = .34, *P* < .001), trust (*r* = .27, *P* < .001), and provider recommendations (*r* = .12, *P* = .005).

### Perceptions on Screening

There was no significant racial difference for provider recommendations (*t*[557] = −.55, *P* = .583; Black American: 4.95 ± 2.83 vs White American: 5.08 ± 2.26). However, Black Americans perceived marginally greater insurance coverage for screening (*t*[557] = 1.79, *P* = .075; Black American: 6.97 ± 3.01 vs White American: 6.43 ± 2.84). Black Americans, however, did convey significantly lower trust in screening accuracy (*t*[557] = −2.02, *P* = .045; Black American: 6.73 ± 2.56 vs White American: 7.20 ± 2.19) compared with White Americans.

## Discussion

From our study, we discovered that willingness to be screened did not differ significantly on the basis of race. This finding is similar to previously published literature by our research team showing that lung cancer screening receptivity was comparable in Black and White Americans.^24^ There was significantly lower trust among Black Americans in the accuracy of screening results, however. Systemic discrimination and inequities have created a deep lack of trust of the health care system by Black Americans. The term “Black iatrophobia” describes the fear of medical institutions by Black Americans from decades of mistreatment and unfairness.^25^ This mistrust continues to be a barrier to increased screening rates among Black Americans. Strategies to overcome this mistrust include assembling more diverse caregiver teams and creating effective communication strategies when interacting with community residents. If these barriers can be reduced, then racial disparities in lung cancer screening may be reduced, as it appears that there is equal receptivity to be screened.

The present study examined individual, provider, and structural barriers to lung cancer screening among a diverse community sample. The study assessed whether these barriers—and their relationship to screening willingness—differed by race, given existing racial disparities in lung cancer screening.^26^ To reach this diverse community sample, we engaged participants in familiar community sites rather than clinical settings. Locations were intentionally selected to reach diverse groups of adults in settings where they naturally gather and feel at ease. Although an event like a baby shower may not seem appropriate, our team discovered that there was a significant ease and comfort level of participants at this type of event. Specifically, there was a multigenerational cross-section of the community that were immersed in a joyous and nonanxious environment. The Hawthorne effect describes an alteration in behavior by individuals who are aware that they are being observed. We believed that community and social events would help to mitigate this effect. Investigators at medical centers should be encouraged to “think outside the box” when planning outreach events designed to reach a culturally diverse part of their surrounding community.

There is a tremendous opportunity for surgeons to increase the overall participation rate of lung cancer screening. Surgeons have the unique perspective of interacting with primary care physicians, oncologists, and pulmonologists. Surgeons also have been intimately involved in the evolution of lung cancer guidelines for decades and are acutely aware of the benefits of screening. There are several interventions that we would recommend to surgical groups to adopt. First, surgeons should be encouraged to attend community events personally and speak about the benefits of lung cancer screening. Our team routinely visits faith-based locations and community health fairs with patients who have consented and volunteered their time. During these outreach visits, the patients have given testimonials about their real-life positive experiences with screening. When these testimonials are paired with input from the surgeon about the patient's care and outcome, we have seen an impressive level of favorability and subsequent uptake of screening by community members. Second, surgeons should work with their electronic health record administrators to create alerts for providers on patients who are eligible.^27^ In addition, surgeons should work with these administrators to ensure that smoking history is accurate and present on as many patients as possible. Unfortunately, previous literature has suggested that electronic health records lack sufficient documentation to determine eligibility for lung cancer screening in more than 30% of all patients.^28^

This design builds on our investigative team's previous literature regarding medical mistrust and its effect on lung cancer screening.^24^ By selecting familiar and highly frequented community sites, the intent was to create an environment suitable for engagement and reduce potential uneasiness related to the traditional hospital setting.^29^, ^30^, ^31^ Select members from the research team also had previous familiarity with these locations, acting as liaisons between the team and the religious organizations in planning these events. In discussing with community members in the process of surveying, the team was able to share data regarding lung cancer screening as a means of contextualizing the study and its purpose. This conversational approach was used by many research team members to give an initial background for the survey and simultaneously promote the understanding of lung cancer screening in the Detroit community. Although there was no cross comparison in this particular study, we did find an overall sense of ease and interest when we approached individuals in these familiar community-based locations. As medical institutions design processes to increase lung cancer screening, we advise that navigators and directors create opportunities to interact with the surrounding population in community-based locations. Events such as community town halls, faith-based fairs, and festivals will likely lead to an increase in awareness and involvement by individuals for lung cancer screening.^32^^,^^33^ Many institutions already conduct similar events, but they potentially could be enhanced with experiences like the mutual patient-surgeon testimonial described earlier.

Another observation from our study was that we were able to recruit a diverse and large number of participants to complete the survey. We encountered a very high level of eagerness among community residents to be screened and an overall pleasant response to being approached by our research team. Most current and previous literature have identified a considerable lack of racial diversity in most clinical research trial.^34^^,^^35^ Our study, by contrast, had a Black American participation rate of 78%. In addition, we were able to recruit a large number of participants easily compared with performing the study within the hospital or through mass emailing. Future studies that seek a diverse and sizeable cohort may benefit from engaging individuals in community-based locations.

Our team intentionally included all adults, even those who were pre-eligible. Numerous studies have shown that men consistently underuse preventive health care services compared with women.^36^^,^^37^ One possible reason is that guidelines for screening tend to be at a younger age for women compared with men. As women are recommended for cancer screening for diseases such as cervical cancer by age 21 years, women may be more familiar and comfortable with screening. Our previous publication by our collaborative team examined perspectives of lung cancer screening in young population (ages 30-49 years) to examine attitudes in the pre-eligible population.^24^ Although previous literature has focused on attitudes in adults who are currently eligible for lung cancer screening, we believe there is utility in further examination of viewpoints in pre-eligible adults as well.

Among the entire study cohort, screening willingness was significantly associated with provider recommendations. Black Americans who are eligible for lung cancer screening are less likely to be referred compared with their White American counterparts.^38^ In addition, our investigative team has shown in previous literature that Black Americans have equal willingness to undergo lung cancer screening once educated about lung cancer risks, prevention and screening recommendations.^8^ These findings underscore the importance of the caregiver in increasing lung cancer screening rates and reducing racial disparities.

There are numerous benefits associated with reducing racial disparities among health care providers.^39^ Creating a more diverse set of providers may allow for equal referral rates and a subsequent decrease in racial screening disparities. Screening willingness was also significantly associated with financial accessibility among the entire cohort. Uninsured individuals tend to have lower rates of cancer screening compared to insured individuals.^40^ Because the percentage of uninsured people may increase in upcoming years, medical institutions should consider programs that may subsidize or eliminate costs of screening for these uninsured members of the community.

There were some limitations to this study. First, we lacked a direct comparison group recruited in clinical settings, which limits our ability to attribute differences in willingness or barriers specifically to the community engagement context. Also, we included many participants who were pre-eligible for screening and did not currently meet the guidelines. It is unclear whether these findings would persist in a cohort that is currently eligible for screening, but we believe that it is important for physicians to understand the attitudes in young adults even if they are not currently eligible. Nevertheless, we believe that strategies to increase awareness among young people will ultimately help to increase uptake of lung cancer screening once eligible. Also, there was a practical reason to include younger adults. As we aim to have numerous centers initiate community engagement efforts to increase screening uptake, we believe that those initial efforts will be more successful by targeting the community broadly. As the relationship with the community strengthens in the future, more targeted communication efforts can be conducted. In addition, we decided to include only Black and White participants. We believed that increasing the number of groups would decrease our statistical power, given the number of participants. However, the rates of lung cancer screening are low across the entire population, so future studies should include more races and ethnicities. Finally, we had a relatively limited set of questions in our survey. Given that the cash compensation was relatively low, a more comprehensive set of questions would have likely limited the number of participants. We also were concerned about elevating levels of mistrust in the community for the medical community. We believed that a long and involved survey with numerous identifying questions may not be received well by pedestrians that we engaged. We do recognize, however, that there is important information that can be gained with more sociodemographic information. As we continue to increase the comfort of the community with our outreach efforts and team members, we will likely be able to perform more in-depth surveys with more detailed demographic information.

Overall lung cancer screening rates are low, and there are racial disparities that still exist. Engaging individuals in community-based locations appears to be received positively, and allows for a diverse and large number of participants. There appears to be similar willingness to be screened between Black and White races, but Black individuals have less trust in the accuracy of screening results. Future screening efforts should use a multilevel strategy targeting financial accessibility, provider engagement, and trust—tailored to the distinct needs of Black and White Americans—and should leverage community-based outreach as a scalable and effective approach to reach diverse populations.

### Webcast

You can watch a Webcast of this AATS meeting presentation by going to: https://www.aats.org/resources/willingness-to-receive-lung-ca-12374.

**Figure:**
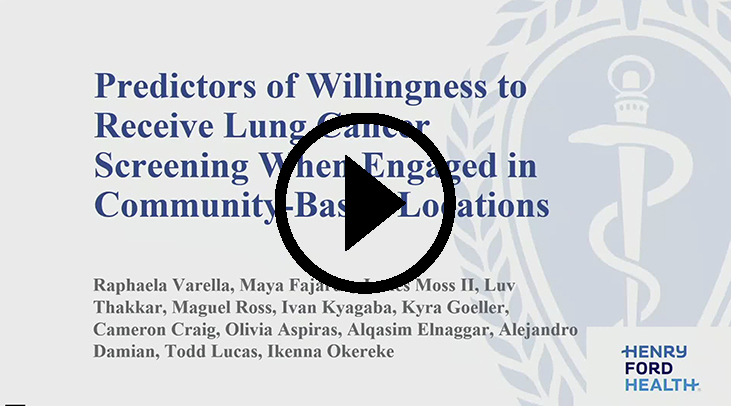

### Audio

You can listen to the discussion audio of this article by going to the supplementary material section below.

## Conflict of Interest Statement

The authors reported no conflicts of interest.

The *Journal* policy requires editors and reviewers to disclose conflicts of interest and to decline handling or reviewing manuscripts for which they may have a conflict of interest. The editors and reviewers of this article have no conflicts of interest.

## Supplementary Data

### Audio

Audio 1 This is an audio recording of the presentation of the associated abstract and discussion. The abstract presentation ends at 6:39 and the discussion begins at 6:52.

Video 1 Video available at: https://www.jtcvs.org/article/S2666-2736(26)00365-7/fulltext.
