# Supplementary material for: Willingness to receive lung cancer screening when engaged in community-based locations
Source: JTCVS Open. 2026 Jun 25;32:101942. doi: 10.1016/j.xjon.2026.101942 (PMC13477103; doi:10.1016/j.xjon.2026.101942)
Supplement: Supplementary file 1 [file mmc2.pdf]

Edited Survey Questions

1. Demographics Questions
    a. What is your language preference?
    b. What is your email?
    c. In what county do you reside?
    d. How old are you in years?
    e. What category best describes your race/ethnicity?
        i. 1= White/Caucasian
        ii. 2= Black/African American

2. **Think of the below ladder as representing where people stand in their communities**. People define community in different ways; please define it in whatever way is most meaningful to you. At the **top rung** of the ladder are people who have the highest standing in their community. At the **bottom rung** are the people who have the lowest standing in their community.

    Where would you place yourself on the ladder? Please select ONE rung via the dropdown menu below where you think you stand at this time in your life, RELATIVE TO OTHER PEOPLE IN YOUR COMMUNITY.

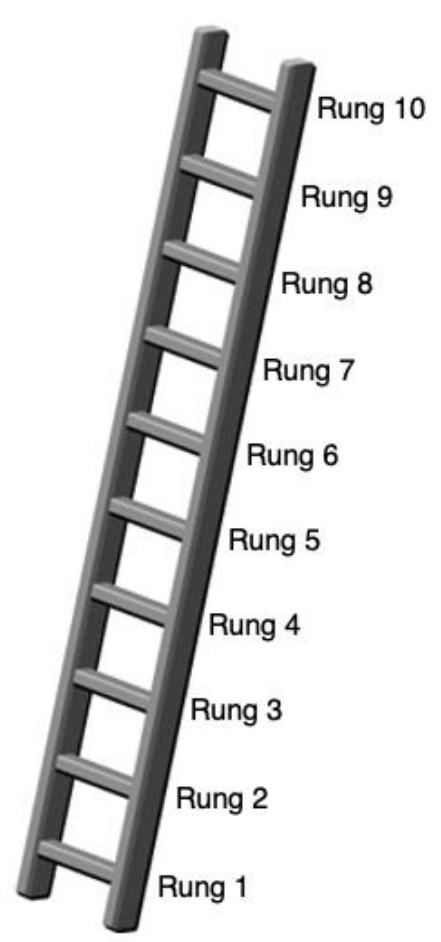

3. **Think of the below ladder as representing where people stand in the United States**.

    At the **top rung** of the ladder are the people who are the best off --those who have the most money, the most education and the most respected jobs. At the **bottom** rung are the people who are the worst off -- who have the least money, least education, and the least respected jobs or no job. The higher up you are on this ladder, the closer you are to the people at the very top; the lower you are, the closer you are to the people at the very bottom. **Where would you place yourself on this ladder?**

Please select <u>ONE</u> rung via the dropdown menu below where you think you stand at this time in your life, <u>RELATIVE TO OTHER PEOPLE IN THE UNITED STATES.</u> Click the "Next" button to the right to continue once, you have made your selection.

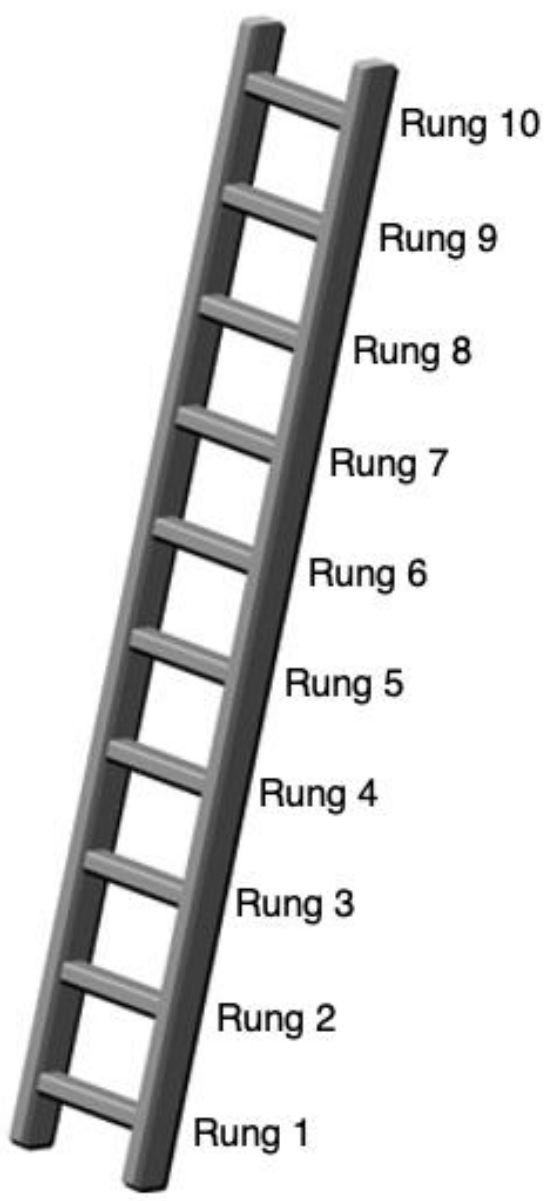

4. Smoking History
    a. Which option best describes you?
        i. 1= I am a current smoker
        ii. 2= I am a former smoker
        iii. 3= I am a non-smoker
    b. You indicated you are a current or former smoker. How many years have you or did you smoke?
        i. - Enter number provided
    c. If you are a former smoker, how many years ago did you quit?
    d. How many cigarettes do you (or did you) smoke per day on average? (1 pack = 20 Cigarettes)
        i. Enter number provided
    e. What was your biological sex at birth?
        i. 1= Male
        ii. 2= Female
5. Which gender do you currently most identify with?
    a. 1= Male
    b. 2= Female
    c. 3= Other
6. Lung Cancer Screening -
    a. How likely are you to pursue lung cancer screening if recommended?

    a. Scale: 1 (Very unlikely) to 10 (Very likely)

  b. Do you believe your medical coverage would be an obstacle to getting lung cancer screening?
    a. Yes
    b. No
  c. Can you afford lung cancer screening if recommended?
    a. Yes
    b. No
  d. Do you have suitable access to healthcare?
    a. 1= No
    b. 2= Yes
    c. 3= Maybe, I'm not sure
  e. Do you think smoking a pipe or E-cigarettes can reduce the risk of lung cancer?
    a. 1=Yes
    b. 2=No
  f. Do you think non-smokers may get lung cancer from exposure to second-hand smoke or radon gas?
    a. 1=Yes
    b. 2=No
  g. Do you think most patients with early lung cancer have symptoms?
    a. 1=Yes
    b. 2=No
  h. Do you think smokers between the ages of 50 and 80 should get lung cancer screening?
    a. 1=Yes
    b. 2=No
  i. Do you think lung cancer screening can be done quickly?
    a. 1=Yes
    b. 2=No
  j. Please select rather the scenario describes your experience or not:
    a. 1= I have read that lung cancer screening could add years to my life and/or help me feel more secure in my health.
      i. 1=Yes
      ii. 2= No
    b. 1= I have read that lung cancer screening may be especially important considering the risk factors associated with my race.
      i. 1= Yes
      ii. 2= No

7. Lung Cancer Beliefs
    a. How likely do you feel that you will get lung cancer?

        i. Choose a number on the scale one being not at all, ten being inevitable
            1. 1-10
    b. How at risk do you think you are getting lung cancer?
        i. Choose a number on the scale one being not at all, ten being inevitable
            1. 1-10
    c. If you were to get lung cancer, how serious of a health problem do you think it would be for you?
        i. Choose a number on the scale one being not at all, ten being inevitable
            1. 1-10
    d. How helpful do you think lung cancer screening is in preventing you from dying form lung cancer?
        i. Choose a number on the scale one being not at all, ten being inevitable
        ii. 1-10
    e. Do you think that your doctors are less likely to offer you lung cancer screening due to your race?
        i. Choose a number on the scale one being not at all, ten being inevitable
            1. 1-10
    f. How likely is it that you will have a lung cancer screening CT scan in your life?
        i. Choose a number on the scale one being not at all, ten being inevitable
            1. 1-10
    g. How likely is it that you will have a lung cancer screening CT scan in the next 12 months?
        i. Choose a number on the scale one being not at all, ten being inevitable
            1. 1-10
    h. Please rate your attitudes about obtaining lung cancer screening.
        i. 1= Strongly Disagree
        ii. 2= Neutral
        iii. 3= Strongly Agree
    i. Do you feel that lung cancer screenings are accessible by members of your community?
        i. 1 = Strongly Disagree
        ii. 2 = Neutral
        iii. 3 = Strongly Agree
8. How safe do you perceive lung cancer screenings to be?
    a. a. 1 = Not safe
    b. 2 = A bit safe
    c. 3 = Extremely safe
9. Do you believe primary care doctors do enough to promote lung cancer screening among patients inside the community?
        i. Yes

        ii. No

10. How important do you believe lung cancer screening is as compared to other health screenings (e.g., mammograms, colonoscopies)?
    a. 1 = Much less important
    b. 2 = About the same importance
    c. 3 = Much more important
11. What level of trust do you hold in the healthcare system to return accurate lung cancer screening results?
    a. 1 = Low
    b. 2 = Moderate
    c. 3 = High
12. Do you believe that lung cancer screening is generally understood by the public?
    a. 1 = Strongly Disagree
    b. 2 = Neutral
    c. 3 = Strongly Agree
13. How comfortable would you be discussing lung cancer screening with your family or your friends?
    a. 1 = Not comfortable
    b. 2 = Somewhat comfortable
    c. 3 = Very comfortable
14. Would you be more likely to do a lung cancer screening if it were free of charge?
    a. 1 = No difference
    b. 2 = Somewhat more likely
    c. 3 = Extremely more likely
15. How much influence can recommendations from your family have on your decision to undergo or not undergo lung cancer screening?
    a. 1 = None
    b. 2 = Some
    c. 3 = A lot
16. Does the potential for a false-positive result have an effect on your decision to get screened for lung cancer?
    a. 1 = None
    b. 2 = Some
    c. 3 = A lot
17. Below Questions are on a scale of 1(strongly disagree)-10(strongly agree)
18. I believe that lack of awareness is a significant barrier to lung cancer screening.
    a. Scale: 1 (strongly disagree) to 10 (strongly agree)
19. I believe that cultural or language barriers impact people’s ability to access lung cancer screening.
    a. Scale: 1 (strongly disagree) to 10 (strongly agree)

20. I have personally been offered lung cancer screening by my healthcare provider.
    a. Scale: 1 (strongly disagree) to 10 (strongly agree)
21. I feel that healthcare professionals should provide more education about lung cancer screening options.
    a. Scale: 1 (strongly disagree) to 10 (strongly agree)
22. I believe that cultural differences should be considered when promoting lung cancer screening in various communities.
    a. Scale: 1 (strongly disagree) to 10 (strongly agree)
23. I believe that early detection of lung cancer through screening can lead to a better quality of life.
    a. Scale: 1 (strongly disagree) to 10 (strongly agree)
24. I believe that a lack of knowledge about lung cancer screening is a significant barrier to people seeking them out.
    a. Scale: 1 (strongly disagree) to 10 (strongly agree)
25. I believe that fear or anxiety surrounding the results of a lung cancer screening is a barrier to pursuing getting one done.
    a. Scale: 1 (strongly disagree) to 10 (strongly agree)
26. Do you think lung cancer screening can minimize your personal risk of dying from lung cancer?
    a. 1 = Not at all
    b. 2 = Somewhat
    c. 3 = Significantly
27. At what level would you rate your knowledge about the eligibility criteria for lung cancer screening?
    a. 1 = Poor
    b. 2 = Average
    c. 3 = Excellent
28. On a scale of 1 to 10, how well do you trust healthcare providers to have your best interests in mind?
    a. Scale: 1 (No trust) to 10 (Complete trust)
29. Do you believe culture plays a role in people's decision to get lung cancer screening?
    a. 1 = Not at all
    b. 2 = Somewhat
    c. 3 = Significantly
30. I believe systemic racism impacts the quality of care people receive in the healthcare system?
    a. Scale: 1 (strongly disagree) to 10 (strongly agree)
31. Do you feel doctors or healthcare workers treat people of your ethnic group differently than others?
    a. Scale: 1 (strongly disagree) to 10 (strongly agree)

32. Do you believe that lung cancer screening could improve public health outcomes within your local community?
    a. 1 = Strongly Disagree
    b. 2 = Neutral
    c. 3 = Strongly Agree
